# Integrated Analysis of Single-Cell RNA Sequencing and Machine Learning Reveals a T Cell-Specific PANoptosis Signature Predicting Prognosis and Immunotherapy in Prostate Cancer

**DOI:** 10.1155/humu/8889021

**Published:** 2025-11-14

**Authors:** Hua Wang, Wenjin Li, Weiming Deng, Jianjie Wu, Ke Li, Xi Huang

**Affiliations:** ^1^Department of Urology, The Third Affiliated Hospital, Sun Yat-sen University, Guangzhou, Guangdong, China; ^2^Department of Nutrition, The Second Affiliated Hospital, Hengyang Medical School, University of South China, Hengyang, Hunan, China; ^3^Department of Urology, The First Affiliated Hospital, Hengyang Medical School, University of South China, Hengyang, Hunan, China; ^4^Department of Ultrasound, Sun Yat-sen Memorial Hospital, Sun Yat-sen University, Guangzhou, Guangdong, China

**Keywords:** immunotherapy, machine learning, PANoptosis, prognostic signature, prostate cancer, single-cell RNA sequencing

## Abstract

**Background:**

Prostate cancer (PCa) ranks among the most prevalent malignancies, with prognosis heavily influenced by diagnostic stage. The role of PANoptosis in T cell-based immunotherapy has garnered growing attention recently. This study is aimed at establishing a T cell-specific PANoptosis signature (TSPS) to predict prognosis and immunotherapy response in patients with PCa.

**Methods:**

Single-cell RNA sequencing (scRNA-seq) data from the GSE185344 dataset were used to identify T cell-specific genes. A comprehensive machine learning pipeline incorporating 10 distinct algorithms was employed to construct a consensus prognostic TSPS.

**Results:**

The scRNA-seq analysis identified T cells as the predominant cell type, and cell–cell communication analysis indicated heightened activation of specific immune-related signaling pathways in PCa. A consensus prognostic signature comprising nine key genes was developed, demonstrating superior predictive accuracy for clinical outcomes compared to conventional clinical variables. A TSPS-based nomogram was also constructed, displaying strong predictive capability for survival outcomes in patients with PCa. Patients in the high-risk group exhibited greater intratumor heterogeneity, increased immune infiltration, and higher immunosuppression scores, suggesting reduced immunotherapy benefits. Validation with four independent immunotherapy cohorts verified that patients in the low-risk group exhibited more favorable immunotherapy responses. Additionally, 18 compounds were determined as therapeutic options for high-risk patients with PCa. In vitro experiments demonstrated that *UBB* expression was upregulated in PCa, and *UBB* knockdown significantly inhibited PCa cell proliferation and invasion.

**Conclusion:**

We established a consensus prognostic TSPS for PCa, offering a potential foundation for future personalized approaches in risk stratification, prognostic evaluation, and treatment selection for patients with PCa.

## 1. Introduction

Prostate cancer (PCa) remains the second most commonly diagnosed cancer among men, accounting for over 1,466,680 new cases and 396,792 deaths worldwide annually [1]. While the 5-year survival rate for patients with locally advanced PCa undergoing radical surgery exceeds 90%, this rate drops to below 30% for those with distant metastatic disease, despite comprehensive treatment efforts [2, 3]. For advanced PCa, androgen deprivation therapy is the standard approach; however, resistance frequently develops, resulting in metastatic castration-resistant PCa, a condition that remains treatable yet incurable [4]. The high heterogeneity, complex cellular composition, and intricate pathogenesis of PCa present substantial challenges in identifying effective therapeutic targets [5]. Despite the clinical utility of prostate-specific antigen (PSA) screening, its low specificity for clinically significant disease leads to overdiagnosis and overtreatment [6]. Tissue-based markers such as PCA3 and TMPRSS2:ERG improve specificity but lack robust prognostic value and have not been widely adopted for therapy guidance [7]. Furthermore, many emerging molecular signatures show promise in retrospective cohorts but fail to predict treatment response prospectively, highlighting issues with reproducibility and cohort heterogeneity. Consequently, the discovery of novel biomarkers and therapeutic strategies is urgently needed to address these critical clinical challenges.

Programmed cell death (PCD) plays a pivotal role in various physiological and pathological processes, notably in host defense against pathogen invasion [8, 9]. Among the different forms of PCD, the interplay among pyroptosis, apoptosis, and necroptosis has led to the identification of PANoptosis [10, 11], a complex inflammatory cell death pathway that extends beyond any individual PCD mechanism. PANoptosis has been increasingly recognized for its involvement in cancer progression and immune responses [12, 13]. For instance, IRF1 can promote PANoptosis to suppress tumorigenesis, whereas ADAR1 inhibits PANoptosis to support tumor growth [14, 15]. Accumulating evidence implies that PANoptosis exerts vital functions in various biological processes associated with cancer progression, such as immunotherapy response and chemotherapy resistance [16, 17]. Gaining deeper insights into the molecular mechanisms underlying PANoptosis is essential for advancing therapeutic strategies for patients with PCa.

Advancements in single-cell RNA sequencing (scRNA-seq) have enabled detailed characterization of the tumor microenvironment (TME) at cellular resolution [18]. Identification of gene expression profiles within immune cells through scRNA-seq has emerged as a critical tool for predicting the prognosis and immunotherapy response of patients with cancer [19, 20]. T cells are widely recognized as important mediators of immunity, and their state can be influenced by the surrounding TME, potentially affecting immunotherapy efficacy [21]. Meanwhile, machine learning methods provide powerful tools to handle high-dimensional transcriptomic data and identify robust predictive features [22]. By integrating scRNA-seq with bulk transcriptomic data and machine learning, it is now possible to derive predictive models that reflect both cellular heterogeneity and immune contexture in multiple cancers [19, 23, 24]. However, their application in PCa remains limited and requires further investigation. In this study, we aimed to identify T cell-specific genes related to PANoptosis and construct a prognostic signature that could predict both clinical outcomes and immunotherapy responses in patients with PCa using an integrative machine learning pipeline.

## 2. Materials and Methods

### 2.1. Dataset Sources

A brief flowchart of the study design is provided in Figure S1. RNA sequencing (RNA-seq) expression data for patients with PCa were sourced from The Cancer Genome Atlas (TCGA,

https://portal.gdc.com). Additionally, expression profiling data from three external cohorts were acquired, including the GSE70768 (https://www.ncbi.nlm.nih.gov/geo/query/acc.cgi?acc=GSE70768) and GSE94767 (https://www.ncbi.nlm.nih.gov/geo/query/acc.cgi?acc=GSE94767) cohorts from the Gene Expression Omnibus (GEO, https://www.ncbi.nlm.nih.gov/) and the Deutsches Krebsforschungszentrum (DKFZ) cohort from the cBioPortal database (https://www.cbioportal.org/study/summary?id=prostate_dkfz_2018/). Clinicopathological data for patients with PCa from these four cohorts were also collected to construct and validate the TSPS. Additionally, scRNA-seq data from seven paired benign and PCa-enriched prostate tissues were obtained from GSE185344 (https://www.ncbi.nlm.nih.gov/geo/query/acc.cgi?acc=GSE185344). Four immunotherapy datasets—IMvigor210, GSE78220, GSE135222, and GSE91061—were included to evaluate the predictive capability of the TSPS in immunotherapy contexts.

### 2.2. scRNA-seq Data Procession and Analysis

The scRNA-seq data were processed as Seurat objects using the “Seurat” package (Version 5.1.0). Cells were filtered out if they exhibited unique feature counts above 6000 or below 1000, mitochondrial counts exceeding 20%, or ribosomal counts above 40%. Genes expressed in fewer than three cells were excluded. Further identification and removal of doublets and ambient RNA contamination were performed using the “doubletFinder” (Version 2.0.4) [25] and “decontX” (Version 1.0.0) [26] packages. The normalized UMI count data were then multiplied by 10,000 and transformed to a natural log scale. Following normalization, the top 3000 highly variable genes were determined using the variance-stabilizing transformation method. The “Harmony” (Version 0.1.1) package was applied to adjust for batch effects across samples. Principal component analysis (PCA) was conducted on the integrated expression matrix using the RunPCA function, and the top 30 principal components were selected via the FindNeighbors function. Clustering was conducted using the FindClusters function with a resolution of 0.5 for all cells. Two-dimensional visualization was achieved with Uniform Manifold Approximation and Projection (UMAP) using the RunUMAP function. Differentially expressed genes for each cluster were identified using the FindAllMarkers function with |log2FC| and minimum percentage cutoff values set at 0.2. Representative marker genes were selected to classify cell clusters into known lineages.

### 2.3. Cell–Cell Communication Analysis Using CellChat

Additionally, the single-cell gene expression matrix was utilized to model cell-type communication probabilities and to identify significant intercellular communications using the “CellChat” (Version 1.6.1) package with default parameters [27]. Briefly, a normalized Seurat object was used to construct the CellChat object, and CellChatDB.human was chosen as the reference database for ligand–receptor interactions. Communication probabilities were estimated using the computeCommunProb function to evaluate the number and strength of interactions between different cell types. To highlight key interactions within specific signaling pathways, the extractEnrichedLR function was applied to retrieve enriched ligand–receptor pairs and associated signaling genes. A minimum of 10 cells per cell type was required for inclusion, and significant interactions were defined as those with *p* < 0.05.

### 2.4. Identification of T Cell-Specific PANoptosis-Related Genes (TSPRGs)

To identify differentially expressed T cell-specific genes between benign and PCa tissues, gene expression profiles for T cells were extracted from scRNA-seq data, followed by differential expression analysis. This strategy yielded a set of T cell-specific genes that also demonstrated differential expression in bulk PCa tissues, highlighting their potential relevance to both immune cell identity and PCa disease progression [28]. Genes with |log2FC| greater than 0.1 and a *p* value below 0.05 were classified as differentially expressed T cell-specific genes. A total of 976 PANoptosis-related genes (PRGs) were compiled by merging the gene lists for pyroptosis, apoptosis, and necroptosis, with duplicates removed [29]. An intersection of the differentially expressed T cell-specific genes and PRGs was then performed to identify candidate genes, termed TSPRGs. To construct a protein–protein interaction (PPI) network, the human PPI network (minimum required interaction score = 0.4) was obtained from the STRING website (https://string-db.org/), and the Cytoscape (Version 3.10.3) plugin Molecular Complex Detection (MCODE) was applied to detect significant subnetworks using default parameters. Kyoto Encyclopedia of Genes and Genomes (KEGG) enrichment analysis was conducted to explore underlying mechanisms of hub genes in the top two significant models.

### 2.5. Construction and Validation of a Consensus Prognostic TSPS

For the construction of a robust prognostic signature, these TSPRGs were processed through an integrative machine learning pipeline [22]. To construct a robust and generalizable prognostic model, we implemented an integrative machine learning framework involving 10 widely used survival analysis algorithms, including Lasso, Ridge, Elastic Net, Random Survival Forest (RSF), CoxBoost, Survival Support Vector Machine (Survival-SVM), Partial Least Squares regression for Cox models (plsRcox), Stepwise Cox, Supervised Principal Components (SuperPC), and Gradient Boosting Machine (GBM). A total of 101 algorithmic combinations were employed to generate prognostic signatures and optimize prediction models based on 10-fold cross-validation to identify the most valuable model. The TCGA cohort was used as the training cohort, while three independent PCa cohorts (GSE70768, GSE94767, and DKFZ) were used for external validation. Model performance was assessed across all cohorts by calculating Harrell's concordance index (*C*-index), providing a quantitative measure of the predictive accuracy. The model with the highest average *C*-index was selected as the optimal prognostic signature. Subsequently, patients in each cohort were categorized into low- and high-risk groups with the median risk score cutoff. The prognostic value and predictive accuracy of this optimal model were validated using Kaplan–Meier survival analysis and receiver operating characteristic (ROC) curves. Meta-analysis and clinical correlation analyses were conducted to assess the practical clinical applicability of the TSPS.

### 2.6. Independent Prognostic Analysis and Nomogram Development

To determine whether TSPS could serve as an independent prognostic factor for patients with PCa, univariate and multivariate Cox regression analyses were performed. A nomogram was developed for predicting clinical outcomes at 1, 3, and 5 years using the “rms” (Version 6.7.1) package, based on the identified independent indicators. Calibration curve analysis, decision curve analysis (DCA), and time-dependent ROC analysis were conducted to evaluate the accuracy of the nomogram's predictions.

### 2.7. Functional Enrichment Analysis

Gene set enrichment analysis (GSEA) was performed to identify enriched KEGG pathways in low- and high-risk groups through the “clusterProfiler” (Version 4.8.2) and “enrichplot” (Version 1.20.0) packages [30]. Significance was determined using an adjusted *p* value < 0.05, with gene sets containing between 10 and 500 genes retained for analysis.

Additionally, gene set variation analysis (GSVA) was applied to assess functional differences in the HALLMARK gene set between the groups using the “GSVA” (Version 1.48.3) package [31]. Reference gene sets (c2.cp.kegg_legacy.v2023.2.Hs.symbols.gmt and h.all.v2023.1.Hs.symbols.gmt) were downloaded from the Molecular Signatures Database (http://www.gsea-msigdb.org/gsea/msigdb/index.jsp). Pathways with an adjusted *p* value < 0.05 and an absolute *t*-statistic greater than 2.5 were considered statistically significant.

### 2.8. Mutant Allele Tumor Heterogeneity (MATH) Analysis

The MATH score, a tumor-specific metric based on variation in the variant allele frequency across all tumor mutations, was calculated to quantify intratumor heterogeneity for each sample [32]. This score was generated using the “maftools” (Version 2.16.0) package, which facilitates analysis, visualization, and summarization of mutation annotation format files from large cancer cohorts [33]. The prognostic value of MATH in PCa was further assessed via survival analysis.

### 2.9. Tumor Immune Microenvironment (TIME) Analysis

To examine immune microenvironment characteristics in low- and high-risk groups, the “ESTIMATE” (Version 1.0.13) package [34] was employed to compute TME scores, including immune, stromal, and ESTIMATE scores for each sample, with group comparisons conducted using the Wilcoxon test. The “gsva” (Version 1.48.3) package also facilitated single-sample GSEA (ssGSEA) to evaluate immune-suppression pathway activity. The CIBERSORT algorithm was utilized to estimate the relative abundance of each tumor-infiltrating immune cell within PCa samples based on TCGA data [35].

### 2.10. Evaluation of TSPS-Based Therapeutic Benefits

The IMvigor210 cohort and three GEO immunotherapy cohorts (GSE78220, GSE135222, and GSE91061) were used to evaluate TSPS performance in predicting immunotherapy outcomes. Patients were divided into low- and high-risk groups using an optimal threshold value determined by the “survminer” package. A chi-square test was performed to assess differences in immunotherapy response rates between groups. We then retrieved the CTRP (https://portals.broadinstitute.org/ctrp) and PRISM (https://depmap.org/portal/prism/) databases to examine drug sensitivity [36, 37]. Drug sensitivity was assessed using area under the curve (AUC) values, where lower AUC values indicated greater treatment sensitivity. The correlation between gene expression and CTRP drug sensitivity was further explored using the GSCA database (https://guolab.wchscu.cn/GSCA) [38].

### 2.11. Clinical Specimens

A total of 11 cases of benign prostate hyperplasia (BPH) and 49 cases of PCa tissues were collected from The Third Affiliated Hospital of Sun Yat-sen University between 2016 and 2020, with pathological examination confirming each case. The tissues underwent formalin fixation and paraffin embedding. Ethics approval for this study was granted by The Third Affiliated Hospital of Sun Yat-sen University (Approval No. II2024-387-01).

### 2.12. Immunohistochemistry (IHC) Staining and Assessment

IHC followed a standard protocol based on a previous study [39]. A polyclonal rabbit anti-UBB (ubiquitin B) antibody (1:500, Proteintech, United States) and a secondary goat anti-rabbit IgG antibody conjugated with horseradish peroxidase (1:5000, Good-Science, China) were used for staining. Images were captured using an Olympus inverted microscope, and IHC staining was assessed through the immunoreactive score (IRS), calculated as the product of staining intensity and the percentage of stained cells. Staining intensity was scored as follows: negative = 0, weak = 1, moderate = 2, and strong = 3. Staining distribution scores were assigned as 0% = 0, < 10% = 1, 11%–50% = 2, 51%–80% = 3, and > 80% = 4.

### 2.13. Cells and Cell Culture

The following human prostate cell lines were used: LNCaP (prostate adenocarcinoma; RRID:CVCL_0395), 22Rv1 (prostate adenocarcinoma; RRID:CVCL_1045), DU145 (prostate adenocarcinoma; RRID:CVCL_0105), PC-3 (prostate adenocarcinoma; RRID:CVCL_E2RM), and BPH1 (benign prostatic hyperplasia; RRID:CVCL_1091). All cell lines were purchased from the American Type Culture Collection (ATCC, Manassas, Virginia, United States). Cell line identity was verified by short tandem repeat (STR) profiling and showed a 100% match to the corresponding ATCC reference profiles. To ensure culture integrity, cells were screened for mycoplasma contamination on a biweekly basis (every 2 weeks). BPH1, LNCaP, 22Rv1, and PC3 cells were cultured in RPMI 1640 medium (Gibco, United States) supplemented with 10% fetal bovine serum (Gibco, United States), while DU145 cells were maintained in DMEM with 10% FBS. All cell lines were incubated in a humidified environment at 37°C with 5% CO_2_.

### 2.14. Transient Transfection of PCa Cells

A small-interfering RNA (siRNA) kit for UBB knockdown was obtained from RiboBio (Guangzhou, China). Two effective siRNA sequences were used: siUBB#1 (5⁣′-CCGTACTCTTTCTGACTACAA-3⁣′) and siUBB#2 (5⁣′-CCTGCGTCTGAGAGGTGGTAT-3⁣′). siRNA oligonucleotides were transfected into PCa cells with Lipofectamine 3000 reagent following the standard protocols.

### 2.15. RNA Isolation and RT-PCR

For RNA extraction, the HP Total RNA Kit (R6812-02, Omega, United States) was utilized, followed by cDNA synthesis using the RevertAid First Strand cDNA Synthesis Kit (Thermo Scientific, United States). RT-PCR was conducted using a Mastercycler ep realplex PCR system (Eppendorf, Germany) with SYBR Premix Ex Taq (TaKaRa, Japan). Primer sequences were as follows: UBB, forward 5⁣′-GGTGAGCTTGTTTGTGTCCCTGT-3⁣′, reverse 5⁣′-TCCACCTCAAGGGTGATGGTC-3⁣′; GAPDH, forward 5⁣′-CGACCACTTTGTCAAGCTCA-3⁣′, reverse 5⁣′-AGGGGAGATTCAGTGTGGTG-3⁣′.

### 2.16. Western Blotting

Total protein extraction followed the protocol from our previous study [40]. The antibodies used included rabbit polyclonal anti-UBB antibody (1:1000, Proteintech, United States), rabbit monoclonal anti-GAPDH antibody (1:4000, Cwbiotech, China), and horseradish peroxidase–conjugated goat anti-rabbit IgG antibody (1:5000, Good-Science, China). Protein bands were visualized using an ECL kit (KF8005, Affinity, United States) and detected using the Tanon-5200 system. Band density was quantified using ImageJ software (National Institutes of Health, Bethesda, United States).

### 2.17. Cell Viability Assay

The Cell Counting Kit-8 (CCK8, CK04, Dojindo, Japan) assay was performed by seeding PC3 (5 × 10^2^ cells/well) or DU145 (2 × 10^3^ cells/well) into 96-well plates with 100 *μ*L per well. After a day of incubation, supernatants were replaced with fresh medium containing 10 *μ*L of CCK8 reagent, and plates were incubated for 30 min at 37°C. Absorbance was measured at 450 nm using a microplate reader daily over 5 days.

### 2.18. EdU (5-Ethynyl-2⁣′-deoxyuridine) Incorporation Assay

Cell proliferation was further evaluated using the EdU assay (RiboBio, Guangzhou, China). Briefly, PC3 and DU145 cells were incubated with 50 *μ*M EdU for 2 h, fixed, and stained following the manufacturer's protocol. Nuclei were counterstained with Hoechst (Invitrogen), and the cells were imaged via fluorescent microscopy.

### 2.19. Wound Healing Assay

PC3 and DU145 cells were transfected with UBB siRNA or a negative control for 48 h, and the assay was conducted as described previously [41, 42]. Wound areas were photographed at various time points from the same location.

### 2.20. Tumor Cell Invasion Assay

Transwell cell culture inserts with 8-*μ*m membrane pores, precoated with Matrigel, were used to simulate the in vivo invasion behavior of PC3 and DU145 cells. The assay followed the procedure outlined in our previous study [41]. All experiments were repeated at least twice.

### 2.21. Statistical Analysis

Statistical analyses were performed using R and SPSS 26.0 software (SPSS, Chicago, Illinois, United States). Depending on the data, Student's *t*-test, chi-square test, and Fisher's exact test were employed to evaluate significant differences among subgroups. *p* < 0.05 indicated statistical difference.

## 3. Results

### 3.1. scRNA-seq Analysis of PCa Patients

**Figure 1:**
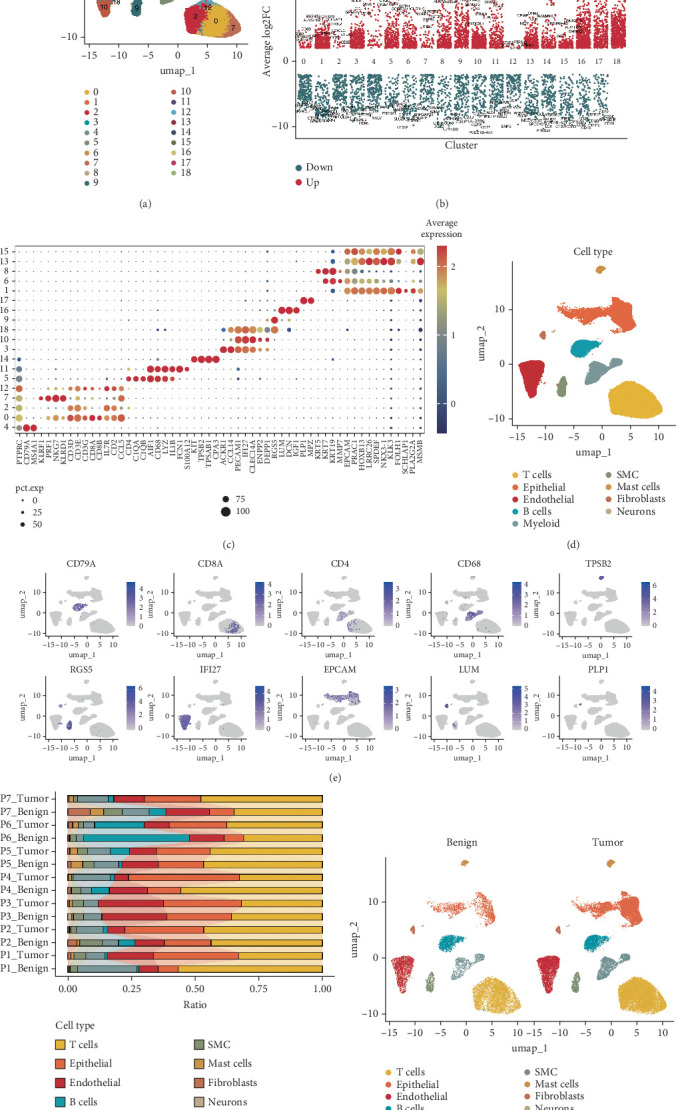
Single-cell RNA sequencing analysis of 44,619 cells from seven paired benign-enriched prostate tissues and PCa tissues. (a) UMAP plot illustrating 18 cell clusters identified across all samples. (b) Differentially expressed genes among the 18 clusters (one vs. others, Wilcoxon test, |log2FC| > 2, adjusted *p* < 0.05). (c) Bubble plot displaying average expression levels of marker genes for each cluster. (d) Annotation of nine major cell types based on marker genes in the UMAP plot. (e) UMAP plots showing representative marker genes for major cell types, color-coded by gene expression levels. Markers included *CD79A*, *CD8A*, *CD4*, *CD68*, *TPSB2*, *RGS5*, *IFI27*, *EPCAM*, *LMU*, and *PLP1*. (f) Stacked bar plots indicating cell fractions of major types in benign-enriched and PCa tissues. (g) Unsupervised graph-based clustering of all samples visualized by UMAP delineated by benign-enriched prostate tissues and PCa tissues.

An integrated illustration of PCa cell types was performed using 10x scRNA-seq data from the GSE185344 dataset, which includes seven paired benign-enriched prostate and PCa tissues. After filtering out low-quality cells, doublets, and ambient RNA contamination, expression profiles for 44,619 cells were obtained. The UMAP plot displayed 18 distinct clusters (Figure 1a). For cell-type annotation, differential gene expression analysis was performed across all scRNA-seq data to identify marker genes for each cluster (Figure 1b). Using canonical marker genes, nine major cell types were identified: T cells, epithelial cells, endothelial cells, B cells, myeloid cells, smooth muscle cells (SMCs), mast cells, fibroblasts, and neurons (Figure 1c,d). The distribution of specific markers across annotated cell types is presented in Figure 1e. Analysis of cell type proportions across 14 patients showed a higher prevalence of T cells in both benign-enriched and PCa tissues (Figure 1f). Additionally, diverse cell types across all samples, distinguished by benign-enriched and PCa tissues, were visualized using UMAP (Figure 1g). Notably, epithelial cells were obviously more abundant in PCa compared to benign-enriched tissues. Although T cell density appeared similar between benign-enriched and PCa tissues, T cells exhibited distinct distribution patterns across paired samples, indicating heightened heterogeneity in PCa tissues.

### 3.2. Cell–Cell Communication Analysis Between Benign-Enriched Prostate Tissues and PCa Tissues

**Figure 2:**
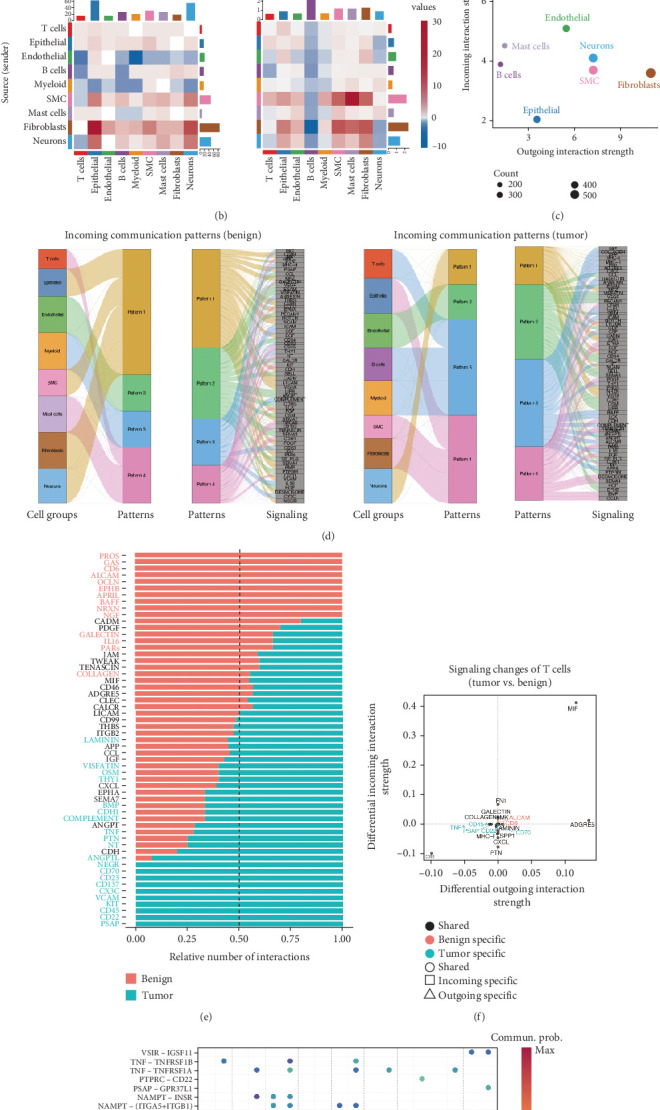
CellChat analysis of the crosstalk between diverse cell types in PCa. (a) Interaction number (left) and interaction strength (right) comparisons between benign-enriched prostate tissues and PCa tissues, with red or blue lines indicating increased or decreased communication in PCa. (b) Heatmaps showing the interaction number (left) and interaction strength (right) in cell–cell communication between benign-enriched prostate tissues and PCa tissues, with red or blue lines indicating increased or decreased communication in PCa. (c) Scatter plot of interaction strength for major cell types in terms of incoming and outgoing signals. (d) The incoming signal pattern and corresponding signaling pathways of major cell types in benign-enriched prostate tissues and PCa tissues. (e) Differences in the number of signaling pathway–related cell communications between benign-enriched prostate tissues and PCa tissues. (f) The intensity changes in the incoming and outgoing signal of T cells between benign-enriched prostate tissues and PCa tissues. (g) Communication probabilities of important ligand–receptor pairs from T cells to other cell types in benign-enriched prostate tissues and PCa tissues, with the dot color reflecting the communication probability.

The CellChat algorithm was utilized to analyze and assess global signaling patterns in cell communication between benign-enriched prostate and PCa tissues, based on single-cell gene expression profiles. Results indicated distinct specificities in cell–cell communication intensity and interaction strength (Figure 2a). For instance, compared to benign tissues, epithelial cells in PCa showed an increase in incoming signal interaction number and strength, while B cells exhibited a marked decrease in interaction strength. Among outgoing signals, fibroblasts displayed significantly elevated signal strength and interaction number, whereas endothelial cells experienced the most pronounced reduction (Figure 2a,b). Notably, T cells demonstrated the highest incoming signal strength across both benign and PCa tissues (Figure 2c).

Further analysis of incoming signaling patterns revealed variations in overall information flow between benign and PCa tissues. In benign tissues, T cells, B cells, and myeloid cells primarily acted as secretory cells, sending signals via Pattern 4, whereas in PCa tissues, T cells and mast cells predominantly sent signals through Pattern 3 (Figure 2d). Communication probabilities highlighted that pathways such as PSAP, CD22, CD45, KIT, and VCAM were more abundant in PCa tissues (blue), while pathways like PROS, GAS, CD6, ALCAM, and OCLN were more prevalent in benign tissues (red) (Figure 2e). Specifically, T cells mediated unique incoming signals (CD22 and CD70) and outgoing signals (TNF, CD45, and PSAP) in PCa tissues, whereas in benign tissues, T cells were associated with specific incoming signaling (ALCAM) and outgoing signaling (CD6) (Figure 2f). Given the central role of T cells in cell communication, ligand–receptor communication probabilities between T cells and other cell types were further analyzed. Findings revealed that TNF-TNFRSF1 ligand–receptor interactions were highly active in communication between macrophages and other cells, showing elevated activity in PCa tissues compared to benign tissues, thereby contributing to immune suppression and PCa progression (Figure 2g). Additionally, predicted interactions such as PTPRC-CD22 and PSAP-GPRC5B were enriched in inferred signaling pathways from T cells to mast cells and neurons in PCa, suggesting potential involvement in shaping the TIME.

### 3.3. Identification of TSPRGs in PCa

To further identify T cell-specific genes in PCa, gene expression profiles of T cells were extracted, followed by differential expression analysis between benign and PCa tissues. A total of 677 T cell-specific genes were identified as differentially expressed, with 303 upregulated in PCa and 374 in benign tissues (Figure S2(a)). PANoptosis has been identified as a possible mechanism influencing both the effectiveness and challenges of T cell-based immunotherapy. All 677 differentially expressed T cell-specific genes were intersected with 976 PRGs, resulting in 67 TSPRGs retained for further analysis (Figure S2(b)). Given the essential role of TSPRGs in the pathways and mechanisms that contribute to PCa, PPI and KEGG analyses were conducted for deeper insights. Using the STRING database (interaction score > 0.4) and Cytoscape software, a complex PPI network with 58 nodes and 440 edges was constructed (Figure S2(c)). The MCODE plugin identified the top two significant modules: Module 1 contained 17 nodes and 106 edges, while Module 2 comprised 13 nodes and 27 edges (Figure S2(d) and S2(e)). Genes within these modules were strongly associated with apoptosis, necroptosis, TNF signaling, and other key pathways implicated in malignancies (Figure S2(f) and S2(g)).

### 3.4. Construction of a Consensus Prognostic Signature Using Integrative Machine Learning Algorithms

**Figure 3:**
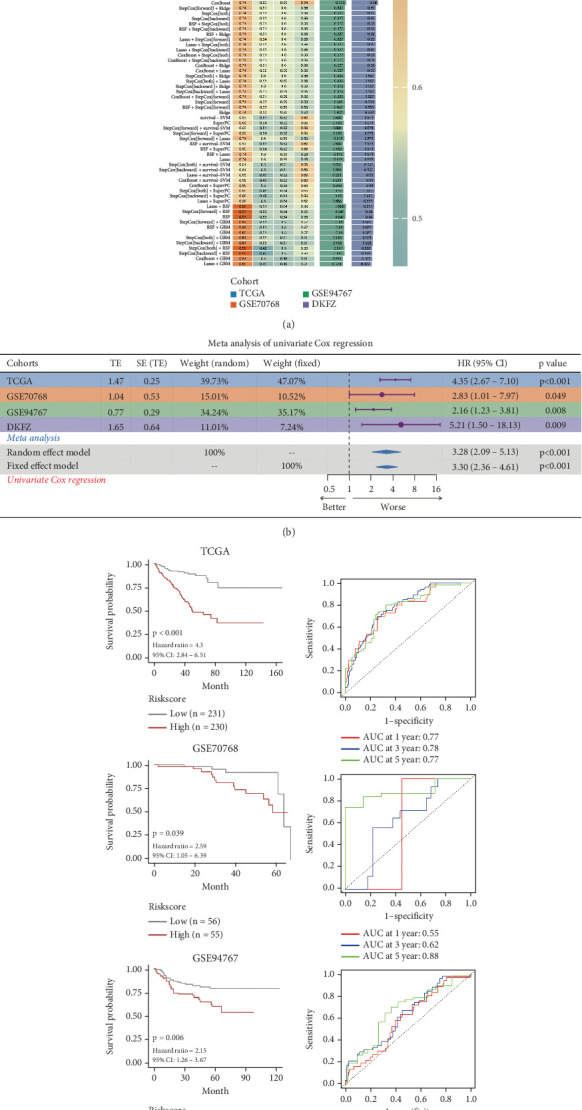
Construction of a consensus prognostic T cell-specific PANoptosis signature (TSPS) for PCa. (a) *C*-index for 101 predictive model combinations using 10 machine learning algorithms in training and validation cohorts. (b) Meta-analysis assessing TSPS association with survival outcomes using univariate Cox regression across TCGA, GSE70768, GSE94767, and DKFZ cohorts. (c) Kaplan–Meier survival curves indicating extended survival in low-risk groups in training and validation cohorts based on TSPS. (d) ROC curves showing 1-, 3-, and 5-year survival prediction accuracy in training and testing sets.

To determine optimal algorithms for constructing a consensus prognostic TSPS for PCa survival prediction, the 67 screened genes were analyzed within an integrative machine learning framework. This study generated 101 models using 10 different algorithms, with the combination of Lasso and plsRcox identified as the optimal model, achieving the highest average *C*-index (0.682) across all model types (Figure 3a). A meta-analysis assessed the association between TSPS and survival outcomes via univariate Cox regression across four cohorts (TCGA, GSE70768, GSE94767, and DKFZ). Results indicated a significant association between TSPS and increased hazard (worse survival) in all cohorts, with hazard ratios (HRs) exceeding 1 in each case. The combined analysis also demonstrated a significant association, supported by *p* values in both random and fixed-effect models, suggesting a consistent effect unlikely due to chance (Figure 3b). Kaplan–Meier survival curves further showed significantly better outcomes for the low-risk group compared to the high-risk group in the TCGA cohort (*p* < 0.001) and similar trends in the GSE70768 (*p* = 0.039), GSE94767 (*p* = 0.006), and DKFZ (*p* = 0.004) cohorts (Figure 3c). Correspondingly, the areas under the ROC curve (AUCs) for biochemical relapse-free survival at 1, 3, and 5 years were 0.77, 0.78, and 0.77 in the TCGA cohort; 0.55, 0.62, and 0.88 in the GSE70768 cohort; 0.59, 0.63, and 0.67 in the GSE94767 cohort; and 0.76, 0.81, and 0.70 in the DKFZ cohort (Figure 3d). These results collectively demonstrate that TSPS provides stable and robust prognostic performance across multiple independent cohorts, underscoring its potential as a reliable prognostic tool for patients with PCa.

### 3.5. Clinical Correlation and Application of TSPS

**Figure 4:**
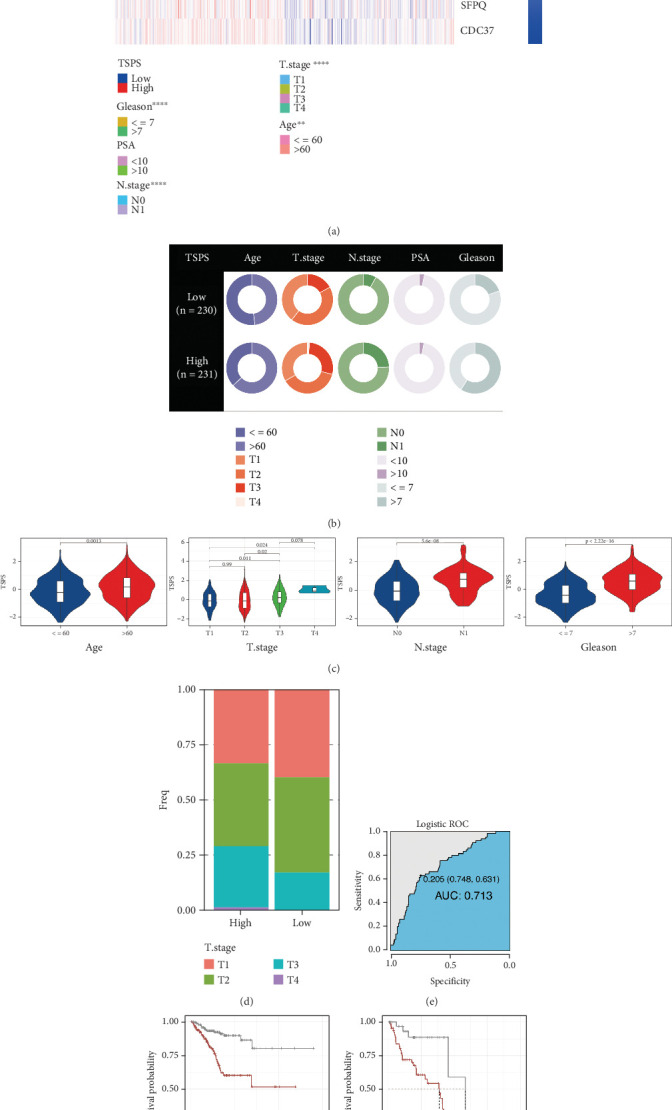
Correlation analysis of TSPS and clinical features in PCa. (a) Heatmap of gene expression profiles and clinical variable distribution in low- and high-risk PCa patients. (b) Pie chart of clinical variable proportions in low- and high-risk groups. (c) Violin plot comparing TSPS distribution across clinical variables (Wilcoxon test). (d) Stacked bar plots showing T stage proportions in low- and high-risk groups. (e) ROC curve predicting node metastasis in PCa patients. (f) Kaplan–Meier survival curves for TSPS in patients stratified by N stage. ⁣^∗∗^*p* < 0.01 and ⁣^∗∗∗∗^*p* < 0.0001.

In clinical practice based on the TCGA cohort, PCa prognosis is typically evaluated based on clinicopathological characteristics, prompting an analysis of TSPS distribution across various clinical parameters in low- and high-risk groups. Significant differences were identified in the distribution of age (*p* < 0.01), T stage (*p* < 0.0001), N stage (*p* < 0.0001), and Gleason score (*p* < 0.0001) between the two groups within the TCGA dataset (Figure 4a,b). Heatmaps further illustrated that *UBB*, *CRIP1*, *FTH1*, *DDIT3*, *TGFB1*, *SFPQ*, and *CDC37* were more highly expressed in the high-risk group, whereas *HERPUD1* and *TPT1* were elevated in the low-risk group. Higher TSPS levels were observed in older men and increased with PCa progression, underscoring a strong association between TSPS and poor prognosis in patients with PCa (Figure 4c). Additionally, TSPS demonstrated predictive potential for the N stage in PCa, achieving an MLR of 0.713 in the diagnostic ROC curve (Figure 4d,e), indicating its potential utility in predicting PCa metastasis. Stratified survival analysis further showed that TSPS effectively distinguished prognosis by N stage, yielding better outcomes for patients with PCa in the low-TSPS subgroup (Figures 4f).

**Figure 5:**
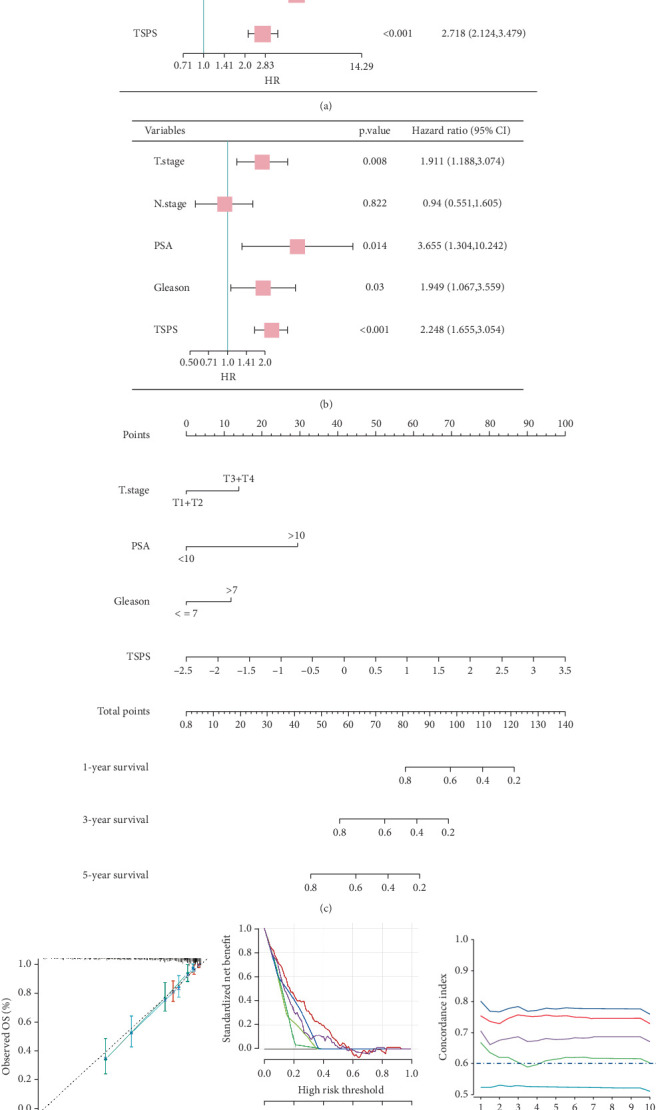
Development and evaluation of the prognostic nomogram. (a) Univariate Cox regression of TSPS and clinical variables in the TCGA cohort. (b) Multivariate Cox regression of TSPS and clinical variables in the TCGA cohort. (c) Nomogram combining TSPS with independent clinical variables in the TCGA dataset. (d) Calibration curves for 1-, 3-, and 5-year survival prediction by the nomogram. (e) Decision curve analysis for 3-year survival prediction. (f) Time-dependent ROC analysis of the nomogram.

Subsequently, univariate and multivariate Cox regression analyses were performed to identify independent prognostic factors in PCa. Univariate analysis, adjusted for conventional clinical variables, identified T stage, N stage, PSA, Gleason score, and risk score as significant outcome predictors (Figure 5a). Multivariate analysis further established T stage, PSA, Gleason score, and risk score as independent prognostic factors for survival outcomes in patients with PCa (Figure 5b). A nomogram was subsequently developed incorporating these four independent indicators, enhancing precision in risk assessment for patients with PCa (Figure 5c). The nomogram's calibration curve demonstrated high stability and accuracy at 1, 3, and 5 years (Figure 5d). DCA confirmed that the nomogram provided the highest decision effectiveness across these time points (Figure 5e), and time-dependent ROC analysis showed that the nomogram outperformed TSPS-based risk scores and other conventional clinical variables alone (Figure 5f).

### 3.6. Molecular Characteristics and Intratumor Heterogeneity of TSPS

Functional enrichment analyses were conducted to further investigate the molecular mechanisms underlying TSPS in PCa onset and progression based on the TCGA cohort. GSEA of KEGG pathways revealed distinct enrichment patterns: the low-risk group was enriched in pathways related to valine, leucine, and isoleucine degradation (Figure S3(a)), whereas the high-risk group demonstrated significant enrichment in antigen processing and presentation (Figure S3(b)). Correlations between TSPS and HALLMARK gene sets are illustrated in Figure S3(c), highlighting specific activations in the high-risk group, including DNA repair, E2F targets, and angiogenesis (Figure S3(d)). Conversely, the low-risk group was linked to pathways such as androgen response, protein secretion, and HEME metabolism. Survival curves confirmed that pathways enriched in the high-risk group correlated with poorer prognosis (Figure S3(e) and S3(f)), indicating distinct molecular characteristics among different risk groups.

To evaluate genomic intratumoral heterogeneity in patients with PCa, MATH scores were calculated, revealing significantly higher scores in the high-risk group, suggesting greater heterogeneity (Figure S4(a)). However, survival analysis showed no notable difference between the low- and high-MATH groups (Figure S4(b)). Further exploration of somatic mutation frequencies showed that, in the high-risk group, *TP53*, *SPOP*, *TTN*, *FOXA1*, and *KMT2D* were the most commonly mutated, while in the low-risk group, the top mutations occurred in *SPOP*, *TTN*, *TP53*, *FOXA1*, and *MCU16* (Figure S4(c) and S4(d)). Analysis of correlations among the top 20 mutated genes revealed a higher incidence of cooccurring mutations in the high-risk group, like *MACF1*-*PCLO* and *MCU16*-*RYR1* (Figure S4(e)). This increased heterogeneity may partially explain the prognostic differences observed between low- and high-risk groups.

### 3.7. Correlation Analysis of TSPS and TIME

**Figure 6:**
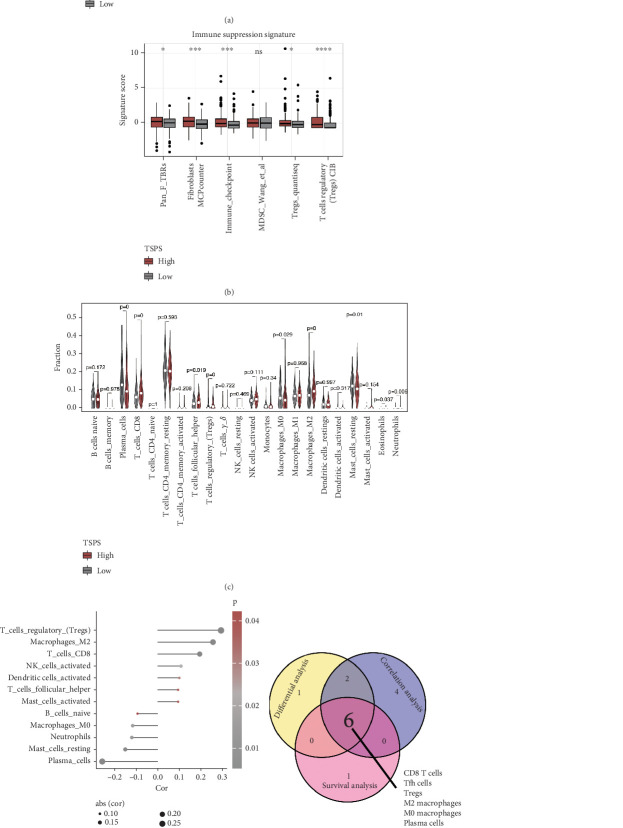
Association of TSPS and immune profiles. (a) Box plots of stromal, immune, and ESTIMATE scores and tumor purity in low- and high-risk groups. (b) Box plots of immune-suppression pathway differences between low- and high-risk groups. (c) Relative infiltration abundance of immune cell subpopulations across risk groups. (d) Correlation between TSPS and immune cell infiltration abundance. (e) Venn diagrams showing intersected immune cell subpopulations from differential expression, correlation, and survival analyses. (f) Correlation analysis of various immune cell types with key signature genes. ⁣^∗^*p* < 0.05, ⁣^∗∗∗^*p* < 0.001, and ⁣^∗∗∗∗^*p* < 0.0001.

Given that the prognostic signature was constructed based on T cell-specific genes, an analysis was conducted to explore the relationship between TSPS and the TIME based on the TCGA cohort. Immune, stromal, and ESTIMATE scores for the TME were calculated from mRNA expression profiles using the ESTIMATE algorithm. Results showed that the high-risk group exhibited significantly higher ESTIMATE, immune, and stromal scores, along with lower tumor purity compared to the low-risk group, indicating greater tumor cell infiltration in the high-risk group (Figure 6a). Additionally, immune status enrichment was quantified using the ssGSEA algorithm, revealing higher activity of immune-suppression pathways such as Pan-F-TBRs, immune checkpoints, fibroblasts, and Tregs in the high-risk group (Figure 6b). A comprehensive assessment of immune infiltration was performed using differential expression analysis, correlation analysis, and survival analysis. Findings demonstrated increased infiltration of CD8+ T cells, T follicular helper (Tfh) cells, T regulatory (Treg) cells, M2 macrophages, eosinophils, and neutrophils in the high-risk group, while plasma cells, M0 macrophages, and resting mast cells showed decreased infiltration (Figure 6c). Correlation analysis identified 12 immune cell types with significant positive or negative associations with TSPS (Figure 6d). Kaplan–Meier survival analysis indicated that plasma cells and M0 macrophages were associated with favorable outcomes in PCa, whereas CD8+ T cells, Tfh cells, Treg cells, M2 macrophages, and monocytes were linked to poor prognosis (Figure S5). Finally, six types of infiltrated immune cells were identified (Figure 6e), with CD8+ T cells, Tfh cells, and Treg cells showing positive associations with most key signature genes (Figure 6f). These findings emphasized the pivotal function of T cells in the construction of TSPS and further confirm their significance within the TIME.

### 3.8. Estimation of the Predictive Ability of TSPS in Immunotherapy and Chemotherapy

**Figure 7:**
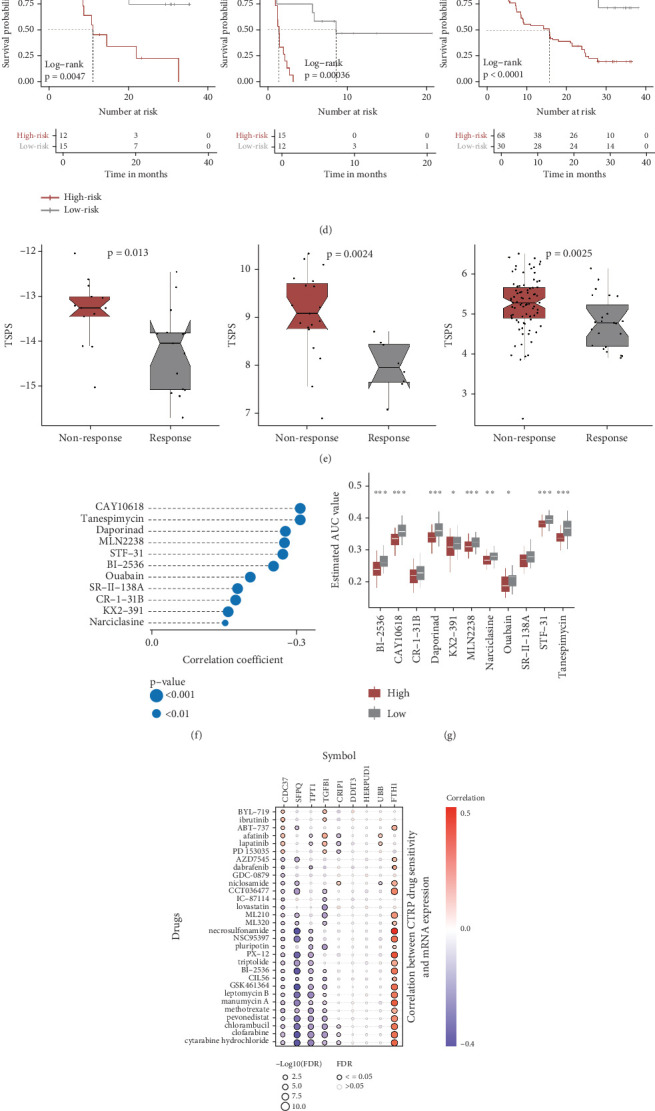
Prediction of immune response and potential therapeutic drugs against PCa based on TSPS. (a) Kaplan–Meier survival curves comparing long-term survival between risk groups in the IMvigor210 dataset. (b) Stacked bar plot of immunotherapy response rates across risk groups. (c) Violin plot showing TSPS distribution across four immunotherapy outcomes. (d) Kaplan–Meier survival curves for PCa patients receiving immunotherapy in GSE78220, GSE135222, and GSE91061 cohorts. (e) Box plots of immunotherapy response distribution in GSE78220, GSE135222, and GSE91061 cohorts by risk group. (f) Correlation analysis between TSPS and 11 CTRP-derived compounds. (g) Comparison of the estimated AUC value of 11 CTRP-derived compounds between the low- and high-risk groups. (h) Correlation analysis between nine critical signature genes and CTRP-derived compounds. (i) Correlation analysis between TSPS and 7 PRISM-derived compounds. (j) Comparison of the estimated AUC value of seven PRISM-derived compounds between the low- and high-risk groups. ⁣^∗^*p* < 0.05, ⁣^∗∗^*p* < 0.01, ⁣^∗∗∗^*p* < 0.001, and ⁣^∗∗∗∗^*p* < 0.0001.

To assess the potential of TSPS in predicting immunotherapy response, four patient cohorts receiving immunotherapy were analyzed. In the IMvigor210 cohort, long-term survival differences were specifically evaluated after 3 months of treatment to account for delayed clinical effects. Results indicated that low-risk patients demonstrated better prognosis, suggesting greater benefit from immunotherapy (Figure 7a). Immunotherapy response distribution analysis further revealed that the low-risk group had a higher proportion of responders (complete response [CR] and partial response [PR]), while the high-risk group contained more nonresponders (progressive disease [PD] and stable disease [SD]) (Figure 7b,c). Similar trends were observed in three other GEO cohorts, with improved prognosis in low-risk patients (GSE78220, *p* = 0.0047; GSE135222, *p* = 0.00036; GSE91061, *p* < 0.0001) (Figure 7d). Correspondingly, responders to immunotherapy showed significantly lower risk scores (GSE78220, *p* = 0.013; GSE135222, *p* = 0.0024; GSE91061, *p* = 0.0025) (Figure 7e).

With the high-risk group showing a limited response to immunotherapy, drug sensitivity and gene expression data from the CTRP and PRISM datasets were used to predict alternative therapeutic options. Based on significant negative correlations (*r* < −0.15) between TSPS and AUC, 11 CTRP-derived compounds were identified, including CAY10618, tanespimycin, daporinad, MLN2238, STF-31, BI-2536, ouabain, KX2-391, narciclasine, SR-II-138A, and CR-1-31B (Figure 7f). Except for SR-II-138A and CR-1-31B, these compounds were predicted to be more effective in low-risk patients (Figure 7g). Based on the GSCA database, further drug sensitivity analysis using correlations with the nine critical signature genes revealed that FTH1 was potentially associated with drug resistance, whereas TGFB1, TPT1, SFPQ, and CDC36 were linked to increased drug sensitivity (Figure 7h). Additional seven compounds from PRISM, including vincristine, VE-822, topotecan, rubitecan, gemcitabine, LY2606368, and dolastatin-10, were also predicted to be effective, with the high-risk group more likely to benefit from these agents (Figure 7i,j). Overall, these results underscore TSPS's potential as a valuable tool for guiding immunotherapy and targeted treatment strategies in PCa, particularly in identifying patients likely to benefit from specific therapeutic agents.

### 3.9. UBB Is Overexpressed in PCa and Drives the Progression of PCa Cells

**Figure 8:**
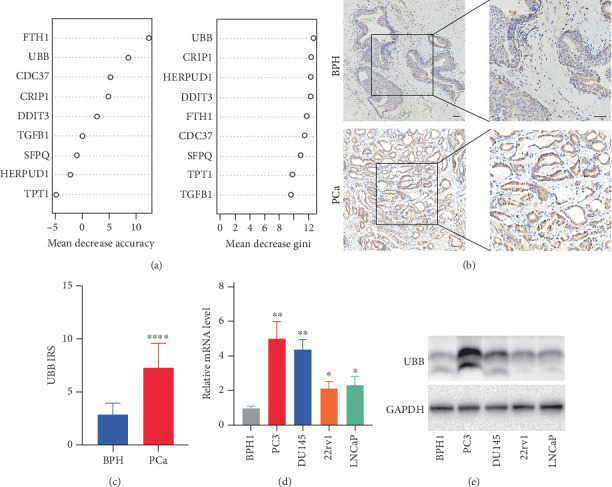
*UBB* expression in PCa and its association with malignancy. (a) Variable importance of the nine signature genes in the TSPS model evaluated by random forest analysis. (b) Representative IHC staining of UBB in BPH and PCa tissues (scale bar = 100 *μ*m). (c) IRS system used for IHC signal assessment between normal and PCa tissues. (d) RT-PCR analysis of UBB mRNA levels in BPH and PCa cell lines. (e) Western blot analysis of UBB protein levels in BPH and PCa cell lines. Data are mean ± standard deviation from three replicates. ⁣^∗^*p* < 0.05, ⁣^∗∗^*p* < 0.01, and ⁣^∗∗∗∗^*p* < 0.0001.

To further evaluate the contribution of individual genes within the signature, we conducted a random forest analysis based on TCGA data. *UBB* demonstrated relatively high importance in survival prediction, with consistently stronger performance than other genes such as DDIT3 and CRIP1 across both MeanDecreaseAccuracy and MeanDecreaseGini metrics (Figure 8a). These findings reinforce the rationale for selecting UBB for experimental validation. Accordingly, *UBB* expression was initially evaluated in 49 PCa tissues and 11 BPH tissues, revealing a significant increase in *UBB* levels in PCa tissues compared to normal tissues (Figure 8b,c). RT-PCR and Western blotting were subsequently used to assess UBB expression in normal (BPH1) and PCa cell lines (LNCaP, 22rv1, PC3, and DU145), with results indicating upregulation of both mRNA and protein levels of *UBB* in PCa cells relative to normal cells (Figure 8d,e).

**Figure 9:**
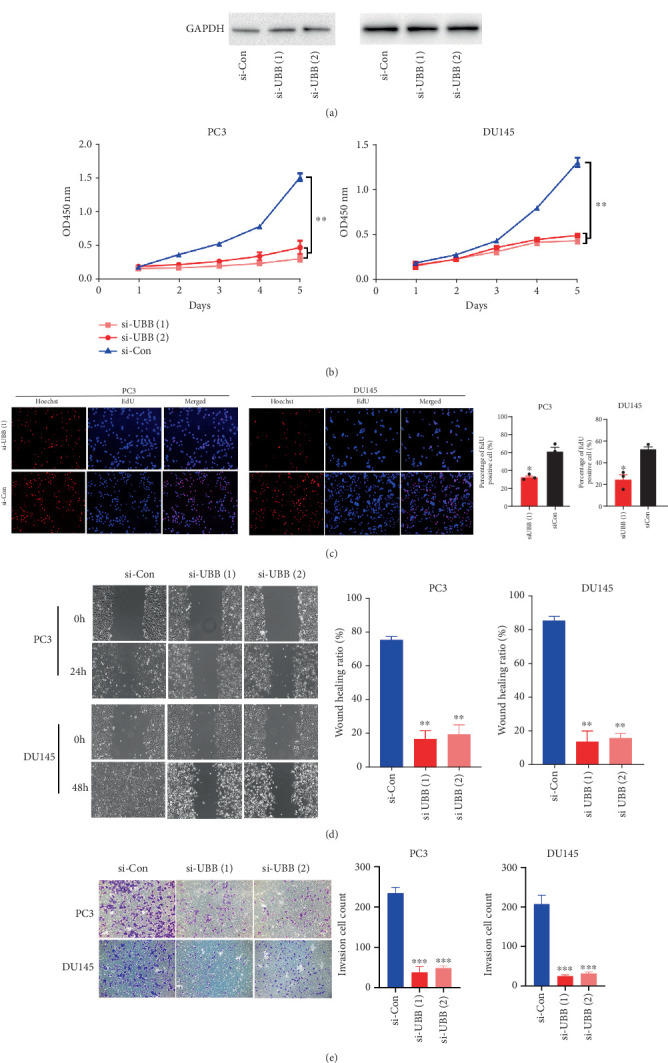
*UBB* promotes PCa cell proliferation and invasion in vitro. (a) Western blot analysis of UBB knockdown efficiency in PC3 and DU145 cells. (b) CCK8 assay to measure the proliferation ability of PC3 and DU145 cells after UBB knockdown. (c) EdU assay showing reduced proliferation of PC3 and DU145 cells after UBB knockdown. (d) Wound healing assay to measure the migration ability of PC3 and DU145 cells after UBB knockdown. (e) Cell invasion assay to measure the invasion ability of PC3 and DU145 cells after UBB knockdown. Data were presented as mean ± standard deviation from three replicates. ⁣^∗∗^*p* < 0.01 and ⁣^∗∗∗^*p* < 0.001.

To explore the role of *UBB* in PCa, functional assays were performed to evaluate the proliferation, migration, and invasion capabilities of PC3 and DU145 cells following *UBB* silencing (Figure 9a). Cell viability assays demonstrated that *UBB* depletion significantly impaired the proliferative ability of PC3 and DU145 cells (Figure 9b). EdU incorporation assays further confirmed these findings from the cell growth curve in both PCa cells (Figure 9c). Consistently, wound healing and invasion assays indicated reduced migration and invasion capacities in PC3 and DU145 cells following *UBB* knockdown (Figure 9d,e). Collectively, these results suggest that *UBB* plays a critical role in PCa progression.

## 4. Discussion

PCa is the most common malignancy among men, ranking third in cancer incidence after lung and colorectal cancers [1]. Despite radical prostatectomy, PCa is marked by high rates of metastasis and relapse, often leading to a poor prognosis [43]. Current PCa classification relies on parameters such as T stage, PSA, and Gleason score for diagnosis [44]. However, the substantial heterogeneity and molecular instability of PCa complicate outcome prediction and therapeutic decisions [45]. Consequently, there is an urgent need to identify novel classification biomarkers for PCa to enhance prognostic accuracy and support individualized clinical therapies.

In this study, scRNA-seq analysis using the GSE185344 database systematically quantified cell-type composition through detected marker genes. T cells emerged as the predominant cell type in both benign-enriched prostate tissues and PCa tissues. Further analysis with CellChat revealed coordinated responses among various cell types [27]. In PCa tissues, T cells drove specific incoming signals (CD22 and CD70) and outgoing signals (TNF, CD45, and PSAP). Among the predicted ligand–receptor pairs, PTPRC-CD22 is known to participate in immune regulation, particularly in T and B cell interactions [46]. PSAP, a secreted glycoprotein, has been associated with tumor progression and neuroimmune communication in various cancers [47]. These interactions may play a role in modulating the immune environment of PCa.

PANoptosis, an inflammatory form of PCD involving pyroptosis, apoptosis, and necroptosis, has recently been implicated in tumor immunity and response to therapy [11]. Given its immunomodulatory role, we explored T cell-specific gene expression related to PANoptosis in PCa. In the present study, gene expression profiles of T cells were analyzed, identifying 677 T cell-specific differentially expressed genes between benign and PCa tissues. After intersecting these with PRGs, 67 TSPRGs were retained to construct a consensus prognostic TSPS through an integrative machine learning approach. The combination of Lasso and plsRcox was chosen to generate the TSPS due to its optimal performance across multiple datasets. Lasso effectively performs variable selection and regularization in high-dimensional genomic data [48], while plsRcox combines partial least squares regression with Cox proportional hazards modeling, enabling robust survival prediction by capturing latent components associated with both gene expression and survival outcomes [49].

The current TSPS comprises nine critical signature genes: *CRIP1*, *FTH1*, *HERPUD1*, *DDIT3*, *TPT1*, *TGFB1*, *SFPQ*, *CDC37*, and *UBB*, many of which have been implicated in PCa development. Among these, *UBB* stands out due to its established role in immune regulation and tumor progression [50]. As a core component of the ubiquitin–proteasome system, *UBB* is involved in T cell receptor signaling, and downregulation of *UBB* ubiquitin levels may be a potential therapeutic intervention for cancer [51]. Furthermore, *UBB* has been linked to modulation of NF-*κ*B signaling and cytokine release [52], which could indirectly shape the recruitment and activity of T cells within the TME. In our study, random forest analysis further confirmed the relatively high importance of UBB in survival prediction, outperforming several other genes in the model. *UBB* was also significantly overexpressed in PCa tissues, and functional validation demonstrated that *UBB* knockdown impaired PCa cell proliferation, migration, and invasion. Collectively, these findings suggest that *UBB* not only plays a role in tumor progression but may also serve as a viable immunomodulatory target in PCa therapy.

Intratumoral heterogeneity is a major challenge that must be overcome in the clinical treatment of cancer patients. Even when tumors initially respond to therapy, resistant subclones present within heterogeneous tumors may repopulate and lead to disease progression [53]. Our analysis revealed that patients in the high-risk group exhibited significantly elevated MATH scores, indicating greater intratumoral genetic diversity. Consistent with our findings, a recent study also demonstrated that PRG expression is closely linked to tumor heterogeneity and can serve as a predictor of both prognosis and immunotherapy outcomes across multiple cancer types [54]. High MATH scores have been previously associated with poor prognosis and aggressive tumor behavior in multiple cancer types [55, 56]. This suggests that increased clonal heterogeneity in the high-risk group may contribute to immune evasion and therapeutic resistance.

Increasing evidence demonstrates that interactions between the TME and immune cells play a critical role in T cell infiltration and immune cell function, significantly influencing immunotherapy outcomes [57, 58]. There was a strong positive correlation between the TSPS-based risk score and immune infiltration, highlighting the genetic–immunological interplay [59]. Specifically, the TME profiling revealed that the high-risk group demonstrated increased infiltration of immunosuppressive cell types (e.g., regulatory T cells and myeloid-derived suppressor cells), along with higher expression levels of immune checkpoint molecules. These findings suggest that the high-risk group is characterized by an immune-excluded or immunosuppressive TME, which may impair effective antitumor immune responses and limit the efficacy of immunotherapy. Survival analysis confirmed that low-risk patients had improved prognosis across four immunotherapy-treated cohorts, suggesting that TSPS may serve as a diagnostic indicator to predict immunotherapy benefit in patients with PCa, with lower TSPS scores indicating greater therapeutic advantage. Importantly, recent insights [60] highlight the crucial role of PANoptosis in reshaping the TIME and sensitizing tumors to immunotherapy, further underscoring the potential relevance of PANoptosis-related signatures like TSPS in guiding treatment strategies. However, given the limited immunotherapy response observed in the high-risk TSPS group, we further explored alternative treatment options using the CTRP and PRISM datasets. Eighteen compounds showed lower AUC values in high-risk patients, indicating potential efficacy. These findings, together with prior evidence supporting PANoptosis-targeted therapies [61], suggest that TSPS may assist in identifying alternative therapeutic strategies for PCa patients less likely to benefit from immunotherapy.

Despite these promising findings, the study has several limitations. First, TSPS was developed using public datasets, necessitating further validation in large-scale, prospective clinical trials. Although we performed in vitro experiments to support the role of UBB, its specific effects on T cell function or PANoptosis pathways remain to be clarified. Moreover, additional studies are needed to validate the biological relevance of other key genes in the TSPS, as their functional roles in PCa progression and immune regulation remain largely uncharacterized. Finally, the immunotherapy-related findings in this study should be interpreted as exploratory; further validation in PCa-specific immunotherapy cohorts is warranted.

## 5. Conclusions

This study identified T cell-specific genes from scRNA-seq data and developed a consensus prognostic TSPS for PCa through an integrative machine learning approach. The TSPS provides a potential foundation for future personalized approaches in risk stratification, prognostic evaluation, and treatment selection for patients with PCa.

## Data Availability

The data that support the findings of this study are available from the corresponding authors upon reasonable request.

## Supporting Information

Supporting Information Additional supporting information can be found online in the Supporting Information section. Figure S1. Overview of the workflow used in the current study. Figure S2. Identification of T cell-specific PANoptosis-related genes (TSPRGs) in PCa patients. Figure S3. Molecular characteristics of TSPS. Figure S4. Mutant allele tumor heterogeneity (MATH) analysis of TSPS. Figure S5. Kaplan–Meier curves for seven immune cell subpopulations.
