# Supplementary material for: Integrated Analysis of Single-Cell RNA Sequencing and Machine Learning Reveals a T Cell-Specific PANoptosis Signature Predicting Prognosis and Immunotherapy in Prostate Cancer
Source: Hum Mutat. 2025 Nov 14;2025:8889021. doi: 10.1155/humu/8889021 (PMC12638157; doi:10.1155/humu/8889021)
Supplement: Supporting Information — Additional supporting information can be found online in the Supporting Information section. Figure S1. Overview of the workflow used in the current study. Figure S2. Identification of T cell-specific PANoptosis-related genes (TSPRGs) in PCa patients. Figure S3. Molecular characteristics of TSPS. Figure S4. Mutant allele tumor heterogeneity (MATH) analysis of TSPS. Figure S5. Kaplan–Meier curves for seven immune cell subpopulations. [file 8889021.f1.docx]

**Supplementary Figures**


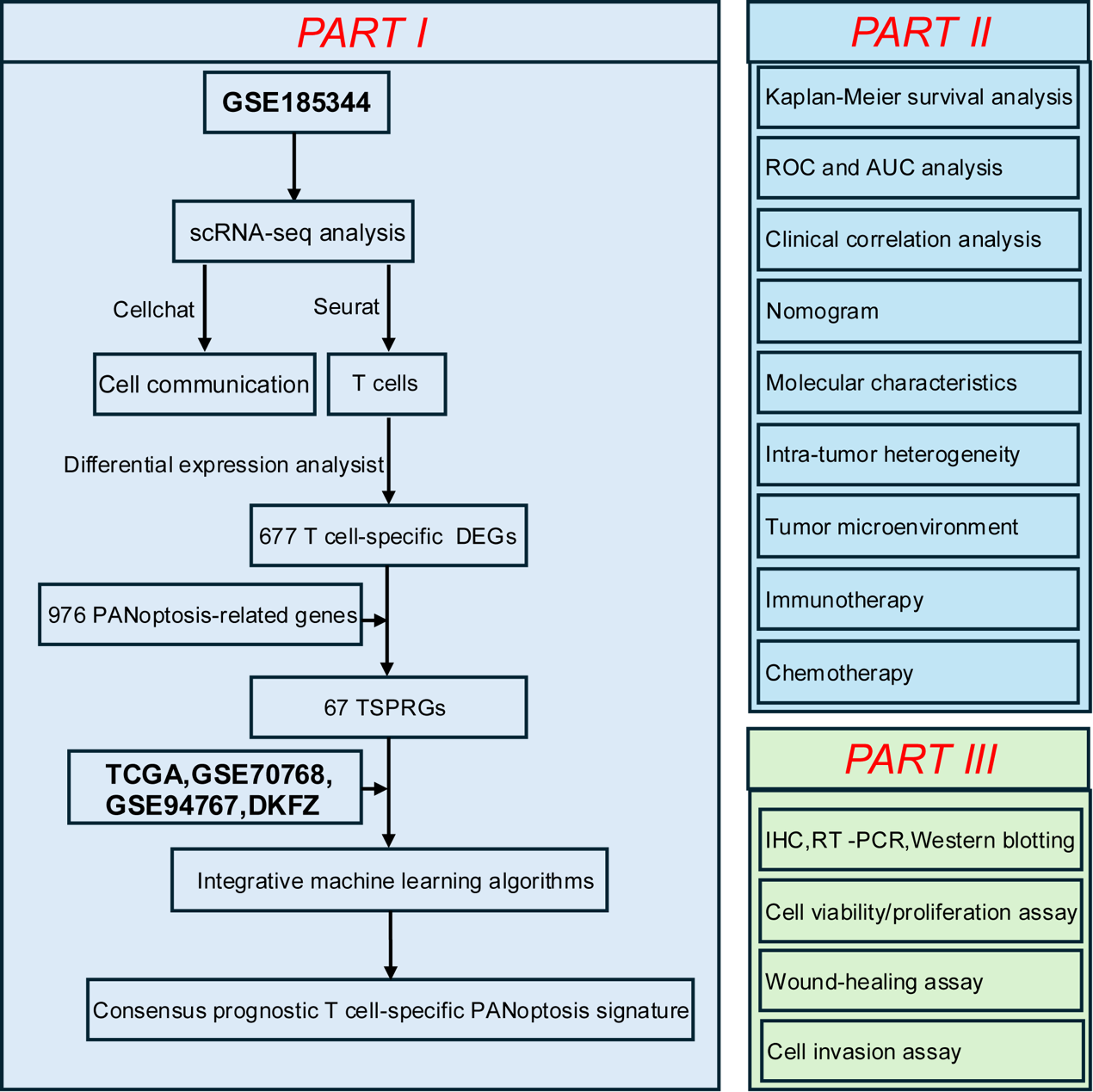


Figure S1. Overview of the workflow used in the current study. PART I: Identification of T cell-specific PANoptosis-related genes and construction of a consensus prognostic T cell-specific PANoptosis signature (TSPS) using integrated analysis of single-cell and machine learning. PART II: Comprehensive evaluation of the clinical utility, molecular mechanisms, and therapeutic response of TSPS. PART III: Validation of *UBB* expression and function in PCa through *in vitro* experiments.


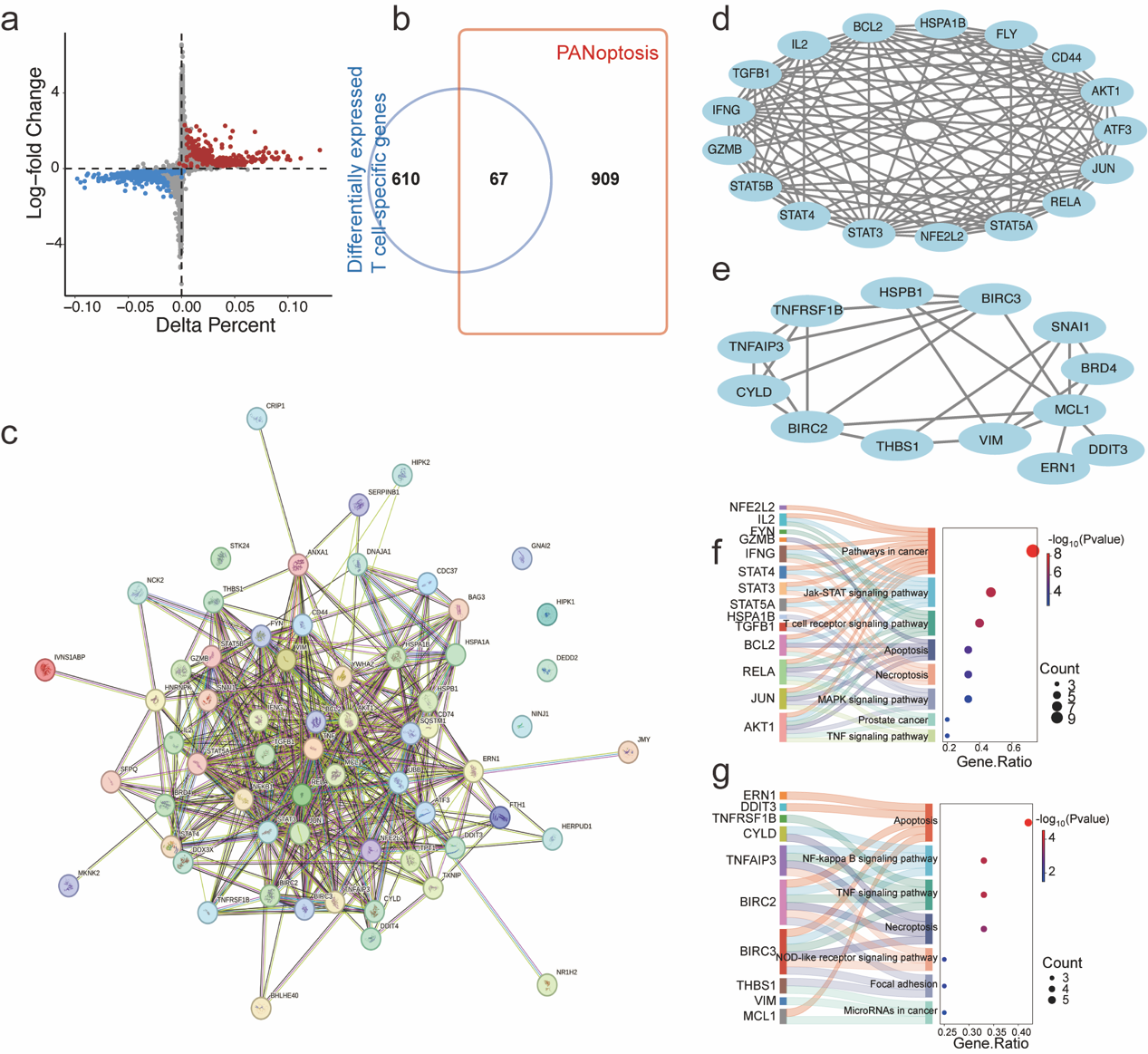


Figure S2. Identification of T cell-specific PANoptosis-related genes (TSPRGs) in PCa patients. (a) Four quadrant plots of T cell-specific differentially expressed genes (DEGs). (b) Venn diagrams showing the 67 intersected TSPRGs of T cell-specific DEGs and PANoptosis-related genes. (c) Protein-Protein Interaction (PPI) network of the 67 intersected genes from STRING. (d) MCODE module 1 and hub gene identification in Cytoscape. (e) MCODE module 2 and hub gene identification. (f) The Kyoto Encyclopedia of Genes and Genomes (KEGG) analysis of the hub gene from Module 1. (g) KEGG analysis of the hub gene from module 2.


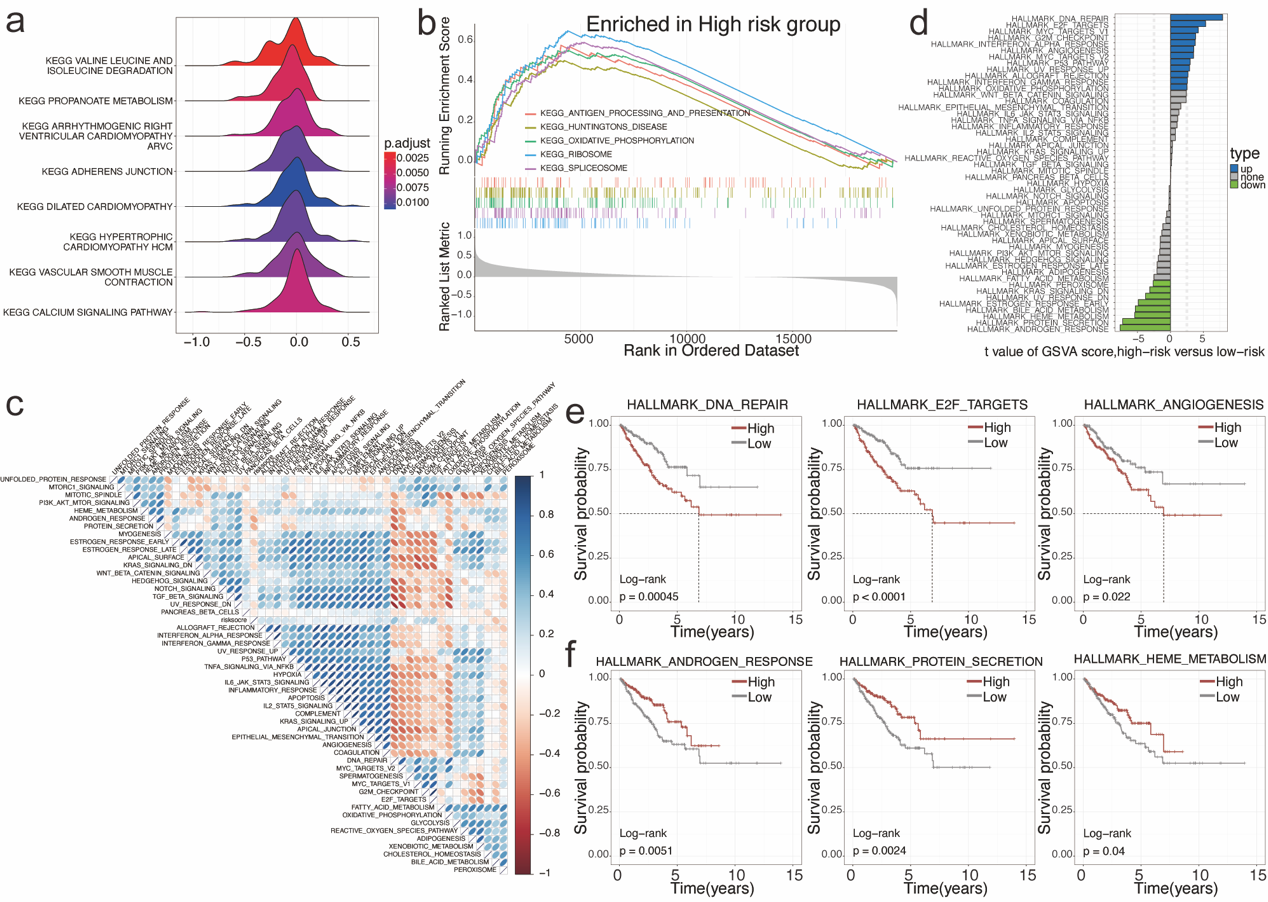


Figure S3. Molecular characteristics of TSPS. (a) Mountain map showing KEGG pathways enriched in the low-risk group based on the gene set enrichment analysis (GSEA). (b) The KEGG pathways enriched in the high-risk group using the GSEA. (c) Correlation analysis of TSPS and hallmark pathways. (d) Differences in the hallmark pathway activities between the low- and high-risk groups. (e) Kaplan-Meier survival curves for selected upregulated hallmark pathways. (f) Kaplan-Meier survival curves for selected downregulated hallmark pathways.


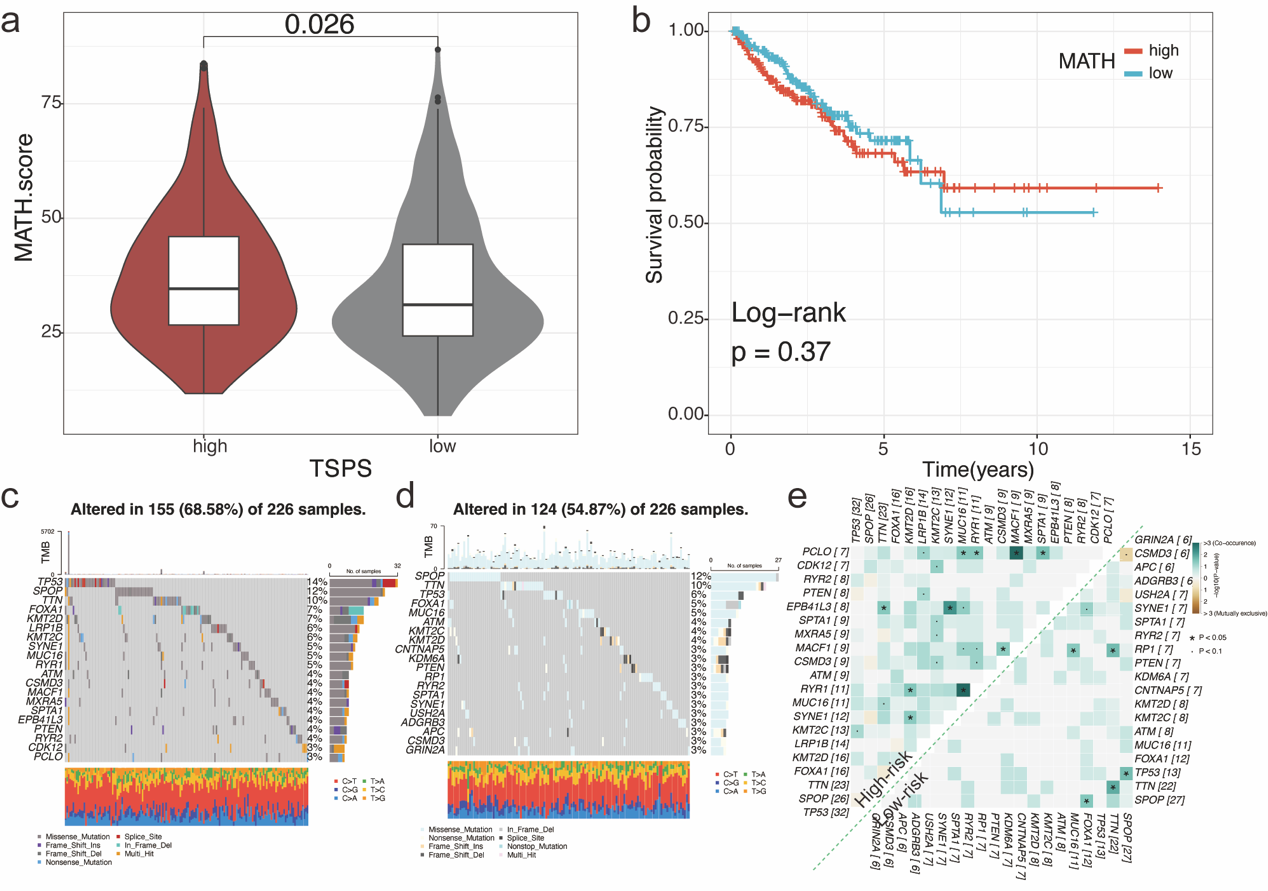


Figure S4. Mutant allele tumor heterogeneity (MATH) analysis of TSPS. (a) Violin plot of MATH differences between low- and high-risk groups. (b) Kaplan-Meier curves for low- and high-MATH groups. (c) Waterfall plot of somatic mutations in the high-risk group. (d) Waterfall plot of somatic mutations in the low-risk group. (e) Mutational exclusivity and co-occurrence in low- and high-risk groups. *P < 0.05.


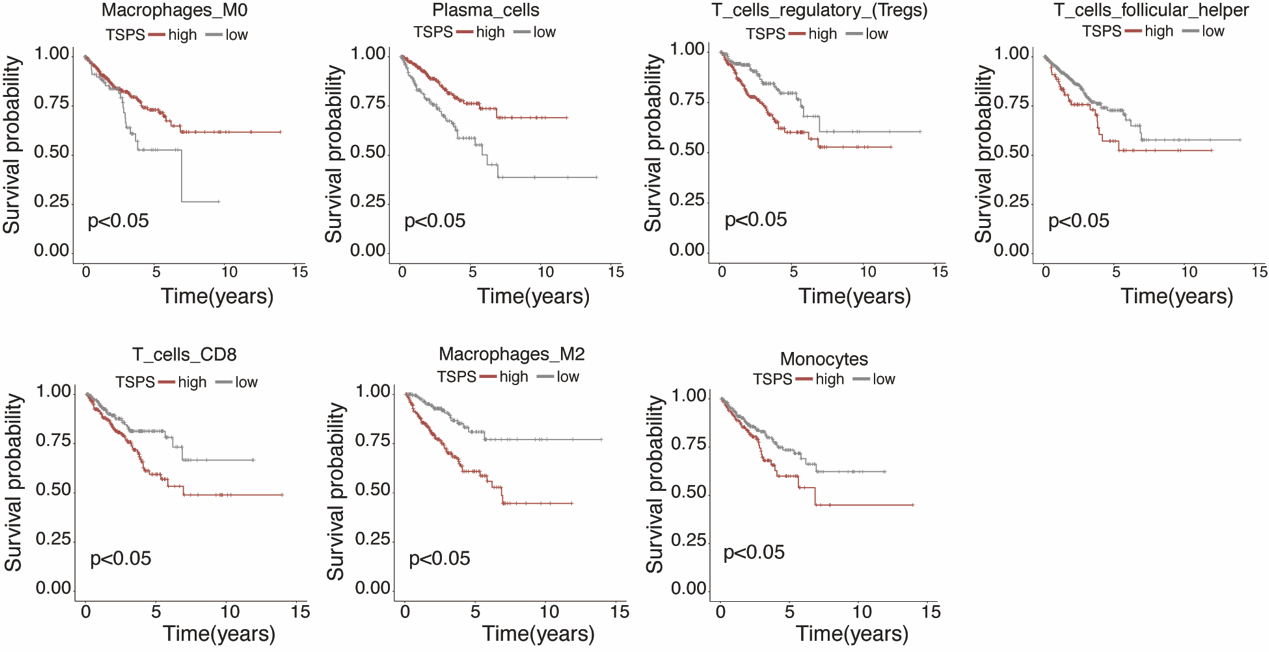


Figure S5. Kaplan-Meier curves for 7 immune cell subpopulations.
